# Tailored radiotherapeutic strategies for disseminated uterine cervical cancer patients

**DOI:** 10.1186/s13014-015-0373-0

**Published:** 2015-04-04

**Authors:** Jung Ho Im, Hong In Yoon, Sunghoon Kim, Eun Ji Nam, Sang Wun Kim, Ga Won Yim, Ki Chang Keum, Young Tae Kim, Gwi Eon Kim, Yong Bae Kim

**Affiliations:** Department of Radiation Oncology, Yonsei Cancer Center, Yonsei University, College of Medicine, 50-1 Yonsei-ro, Seodaemun-gu, Seoul, 120-752 South Korea; Department of Pharmacology, Yonsei University, College of Medicine, Seoul, South Korea; Women’s Cancer Clinic, Yonsei Cancer Center, Yonsei University, College of Medicine, Seoul, South Korea; Yonsei Song-Dang Institute for Cancer Research, Yonsei University, College of Medicine, Seoul, South Korea

**Keywords:** Disseminated uterine cervical neoplasms, Lymphatic metastasis, Visceral organ metastasis, Radiotherapy, Chemotherapy

## Abstract

**Background:**

To investigate the role of radiotherapy (RT) in and to suggest radiotherapeutic strategies for patients presenting with disseminated cervical cancer.

**Methods:**

We retrospectively analyzed 50 patients diagnosed as the disseminated cervical cancer with distant lymph nodal or visceral organ metastasis between September 1980 and August 2012. Patients were divided into two subgroups according to visceral organ metastasis: 35 patients diagnosed with distant lymph node metastasis only (group A) and 15 patients with visceral organ metastasis (group B). All patients received external beam RT to the pelvis (median dose 45 Gy) and high-dose rate intracavitary RT (median dose 30 Gy). Thirty-nine patients (78%) received chemotherapy.

**Results:**

Median follow-up time was 74 months. The 5-year pelvic control rate (PCR) was 85.8%, and the progression-free survival (PFS), and overall survival (OS) rates were 28.7%, and 36.2%, respectively. The major treatment failure was systemic progression (32 patients, 64%). The 5-year PCRs in groups A and B were 87.4% and 74.7%, respectively (*p* > 0.05). Meanwhile, PFS and OS rates for group A were significantly better than those for group B (35.3% vs. 13.3%, *p* = 0.010; and 46.3% vs. 13.3%, *p* = 0.009, respectively).

**Conclusion:**

Our data revealed considerable prognostic heterogeneity in disseminated cervical cancer. Even though a high PCR was achieved in patients treated with definitive RT, survival outcomes were dependent on progression of visceral organ metastasis. Therefore, personalized RT and chemotherapy treatment strategies according to the presence of visceral organ metastasis in disseminated cervical cancer patients may help improve clinical outcomes.

## Background

Stage IVB cervical cancer is defined by the International Federation of Gynecology and Obstetrics (FIGO) as: a carcinoma that has extended beyond the true pelvis and has spread to distant organs. As the FIGO staging system does not differentiate between locations of lymph node (LN) metastasis in stage IVB cancer, clinicians often face diagnostic difficulties, particularly with metastases to the mediastinal, axillary, or supraclavicular LNs. Thus, many clinicians have regarded LN metastasis beyond the extended field (EF) as stage IVB cancer, even in the absence of visceral organ metastasis.

Patients with disseminated cervical cancer tend to have very poor prognosis [1], and the treatment of patients with disseminated cervical cancer tends to vary according to disease characteristics, patient symptoms, and physician preference. However, no consensus has been reached regarding the management of disseminated cervical cancer due to its rarity, and there is a huge heterogeneity in the treatment of disseminated cervical cancer. Nevertheless, while systemic or palliative radiotherapy (RT) for pain, bleeding, or discomfort, is usually recommended [2-6], little is known about the most effective combinations of RT and chemotherapy (CTx) for treating disseminated cervical cancer patients.

Recently, a few reports have suggested that concurrent chemoradiotherapy (CCRT) could increase survival in cervical cancer patients with supraclavicular lymph node (SCLN) involvement [7-9]. Other studies have found that the combined use of RT and CTx with a curative aim, for stage IVB cervical carcinoma, may increase survival [10-12]. Meanwhile, metastasis type has also been reported to be associated with prolonged survival in patients with disseminated cervical cancer [13]. Patients with only lymphatic metastasis had a long-term survival, but patients with hematogenous metastasis showed extremely poor prognosis. Thus, the purpose of this study was to investigate the role of RT in and to suggest radiotherapeutic strategies for treating disseminated cervical cancer patients.

## Methods

### Eligibility

This retrospective study received approval from the institutional review boards (No. 4-2015-0068). We retrospectively reviewed the medical records of patients initially diagnosed with disseminated cervical cancer between September 1980 and August 2012 at Yonsei Cancer Center, Yonsei University College of Medicine, in Seoul, South Korea. The present study included 50 cases of pathologically proven uterine cervical cancer treated with external beam radiation therapy (EBRT) and high-dose rate intracavitary irradiation (ICR). We defined disseminated cervical cancer as follows: evidence of distant LN metastasis beyond the EF (e.g., supraclavicular, mediastinal, axillary, inguinal LNs) or hematogenous metastases to visceral organs. The patients with inguinal LN metastasis with low vaginal involvement were excluded. The diagnosis of disseminated cervical cancer was based on physical examination, diagnostic imaging studies or pathologic findings from biopsy. Patients who presented with LN metastases within the EF or those with recurrent disease, were excluded from the study.

The histological classification of uterine cervical cancer was based on the World Health Organization classification, and FIGO classification was used for clinical staging. The routine procedure for staging included a detailed history review, general physical examination (including inguinal and supraclavicular nodal areas), pelvic examination (including bimanual pelvic and rectal examinations), laboratory tests (complete blood-cell count, serum chemistries, urinalysis), colposcopy, standard chest radiographs, intravenous pyelography, cystoscopy, and sigmoidoscopy. Optional studies (computed tomography (CT), magnetic resonance imaging (MRI), positron emission tomography-computed tomography (PET-CT), or bone scan) were performed to evaluate the extent of disease. The distant spread of tumor was confirmed by imaging studies and/or fine needle aspiration biopsy. In the image interpretation of CT or MRI, the principal criterion for positive node involvement was based on the axial diameter of the LN. LNs larger than 1 cm in the short-axis dimension were considered metastatic node involvement. We also regarded central necrosis as a significant criterion for metastatic disease within the LN [14,15]. In the image interpretation of PET-CT, metastatic LN was defined as follows: (1) the accumulated amount of fluorodeoxyglucose greater than that in the liver or similar to that in the brain cortex, or (2) the standardized uptake value of a lesion that corresponded to the CT and that did not decrease in the delayed PET image compared with the initial value [16]. In principle, it is essential to obtain tissue and pathologically confirm for metastatic lesions. Because we didn’t want to delay definitive treatment due to biopsy or surgical procedures, all the visceral organ and paraaortic lymph nodes metastases were confirmed by only imaging. For patients with palpable neck, axilla, or the inguinal LNs, percutaneous fine needle aspiration biopsy was performed for twenty-one patients at the discretion of the treating physician.

### Treatment protocol

All the patients underwent the RT with definitive aims. EBRT followed by high-dose rate ICR was delivered in all patients. Parametrial, pelvic side wall, or node boost wad performed after EBRT. EBRT was delivered to the whole pelvis or EF through antero-posterior/postero-anterior portals or a four-field box technique, using megavoltage photon beams of ^60^Co or linear accelerators. The superior borders of the whole pelvis and EF, usually L4-L5 and T11-T12 interfaces, respectively, were administered EBRT. The daily fraction of EBRT was 1.8 or 2.0 Gy, administered once daily for 5 days each week. During RT, patients were assessed weekly for cervical tumor response. High-dose rate ICR was delivered via a remote-controlled after loading system. From 1979 to 1989, patients were treated with a ^60^Co source, three times per week at 3 Gy per fraction. After 1989, ICR with ^192^Ir was administered at 5 Gy per fraction, up to a total dose of 30 Gy. Applicator insertion was carried out on an outpatient basis without anesthesia, and each treatment required approximately 10 to 15 minutes. Parametrial, or pelvic side wall boost with central shielding, was administered to patients with persistent parametrial disease, following planned pelvic RT or between brachytherapy sessions. After the patients underwent EBRT with the 45–50.4 Gy dose, response of LNs was evaluated on CT images. If the treatment response of the LNs was not complete remission (non-CR), a node boost was delivered to the multiple fields via the three-dimensional conformal technique. For the para-aortic lymph nodes (PALN), the RT did not exceed 54 Gy, considering the toxicity of the small bowel. In case of RT on the distant LNs or the metastatic bones, EBRT to metastatic sites was administered concurrent with pelvic RT. For the distant LNs, RT was administered with the daily fraction of 1.8-2.0 Gy; and for the metastatic bones, 3 Gy and total of 30–45 Gy. Korean national health insurance does not cover expenses of modern radiation techniques like IMRT or tomotherapy as an initial treatment of cervical cancer which results in huge cost difference bigger than 5 times between 3DCRT and IMRT. Therefore, physicians usually recommend 3D-CRT rather than IMRT or tomotherapy in initially diagnosed cervical cancer these days.

All the patients underwent platinum-based CTx except for 11 patients who were treated only with RT. They were composed of six who had been treated in the 1980s, two elderlies, one with poor general condition, and two patients who refused to undergo CTx. The sequence of RT and CTx was determined upon the physician’s discretion. Platinum single-agent or platinum-based doublet regimens were used. Twenty patients underwent platinum-based single-agent regimens, and 19, platinum-based doublet regimens. CCRT protocol was included two treatment schemes. One scheme was composed of three CTx cycles administered at the beginning of the first, fourth, and seventh weeks of RT. The chemotherapeutic regimens consisted of cisplatin 100 mg/m^2^ or carboplatin 400 mg/m^2^ followed by five consecutive daily infusions of 5-FU 1000 mg/m^2^. Since the publication of randomized trials, weekly chemotherapeutic infusion has been performed with cisplatin (40 mg/m^2^) or carboplatin (AUC, 2) during radical RT [17]. For induction CTx, 100 mg/m^2^ of cisplatin was infused, followed immediately by five consecutive daily administrations of 1,000 mg/m^2^ of 5-fluorouracil, each as a 24-hour intravenous infusion. Other induction CTx regimen was paclitaxel 175 mg/m^2^ and carboplatin AUC 5.

### Follow up and statistical analysis

Treatment responses were evaluated one month after completion of RT via physical examination and imaging studies. Complete remission was defined as the complete disappearance of all measurable disease for at least one month. Partial remission comprised a reduction in lesion diameter of more than 50% with no demonstrable disease progression elsewhere. Stable disease consisted of a decrease in lesion diameter of less than 50% or a 25% increase in lesion diameter without appearance of any new lesion. Progressive disease reflected an increase in lesion diameter of more than 25% with or without appearance of any new lesion. Patients who achieved CR for both the primary cervical masses and metastatic LNs were considered to have achieved overall CR.

Patients were followed-up every 3 months during the second year after completion of therapy, and then every 6 months thereafter. Recurrences in the cervix, vagina, parametrium, or pelvic LNs were defined as pelvic failure. Systemic progression was defined as the appearance of disease in visceral organs or distant LNs, distinct from sites of the original disseminated disease. Overall survival (OS) was calculated from the date of diagnosis to the date of death, or date of last follow-up visit. Progression free survival (PFS) was calculated as the time from the date of diagnosis until the first reported occurrence of tumor progression, or death. The Kaplan-Meier method was used to estimate pelvic control rate (PCR), PFS, and OS. Univariate analysis of risk factors was performed by comparing survival rates using the log-rank test. Multivariate analysis was performed using a Cox proportional hazards model and hazard ratios (HR) with a 95% confidence interval (CI) to identify prognostic factors. A *p-*value of < 0.05 indicated statistical significance.

## Results

### Patient characteristics and treatment profiles

Fifty patients were included in the present study. Patient characteristics and treatment profiles are listed in Table 1. The median age was 50 years (range, 22–77 years). Twelve patients had severe vaginal bleeding which required vaginal packing and blood transfusion. Squamous cell carcinoma was the most common histologic subtype (42 patients, 84%). Sixteen patients (32%) had a hemoglobin level ≤ 10 g/dL before treatment and 36 patients (72%) had tumors measuring > 4 cm in diameter. Thirty-nine patients (78%) received CTx with RT. Patients had received CCRT in 28 (56%), induction CTx in 11 (22%). EBRT was administered to the whole pelvis in 15 (30%) patients, and EF in 35 (70%) patients. The median dose of EBRT to the pelvis was 45 Gy (range, 43.2-54 Gy), and the median ICR dose was 30 Gy. The total dose delivered to point A ranged from 64.5 to 94.25 Gy _EQD2_ (median, 75.5 Gy _EQD2_). Table 2 shows the sites of distant metastases. SCLN was the most common distant metastatic site.

**Table 1:** **Patient characteristics and treatment profile of whole patients and comparison of two subgroups**

**Characteristic**	**Median (range)/no. of patients (%)**			***p*** **-value**
	**Total**	**Group A**	**Group B**	
Age (year)				NS
Median	50	49	51	
Range	22-77	22-74	29-77	
ECOG performance status				NS
0-1	47 (94.0)	34 (97.1)	13 (86.7)	
2-3	3 (6.0)	1 (2.9)	2 (13.3)	
Histopathologic type				0.043
Squamous cell ca	42 (84.0)	32 (91.4)	10 (66.7)	
Adenocarcinoma	5 (10.0)	3 (8.6)	2 (13.3)	
Small cell ca	2 (4.0)		2 (13.3)	
Adenosquamous cell ca	1 (2.0)		1 (6.7)	
Pre-treatment Hb (g/dL)				NS
≤ 10	16 (32.0)	10 (28.6)	6 (40.0)	
>10	34 (68.0)	25 (71.4)	9 (60.0)	
Tumor size (cm)				NS
≤4	14 (28.0)	12 (34.3)	2 (13.3)	
>4	36 (72.0)	23 (65.7)	13 (86.7)	
Dissemination pattern				
Group A	35 (70.0)			
Group B	15 (30.0)			
Treatment type				NS
RT + CTx	39 (78.0)	25 (71.4)	14 (93.3)	
RT alone	11 (22.0)	10 (28.6)	1 (6.7)	
RT field				<0.001
Whole pelvis	18 (36.0)	7 (20.0)	11 (73.3)	
Extended	32 (64.0)	28 (80.0)	4 (26.7)	
RT dose (Gy)				NS
External dose	45.0 (43.2-54.0)	45.0(43.2-52.0)	45.0 (44.0-54.0)	
ICR dose	30.0 (18.0-39.0)	30.0 (18.0-39.0)	30.0 (21.0-30.0)	
Point A dose (EQD2)	75.5 (64.5-94.25)	72.9 (64.5-94.25)	75.5 (67.9-87.5)	

*Abbreviations*: ECOG, Eastern Cooperative Oncology Group; Ca, Carcinoma; Hb, hemoglobin; Group A, Distant lymph node metastasis only; Group B, Visceral organ metastasis; RT, Radiotherapy; CTx, chemotherapy; ICR, intracavitary radiotherapy; Equivalent 2 Gy dose, EQD2.

**Table 2:** **Distribution of disease characteristics**

**Metastatic sites**	**Number of patients**
Distant LN metastasis	
Supraclavicular LN	22
Inguinal LN	6
Mediastinal LN	3
Supraclavicular + mediastinal LN	1
Supraclavicular + axillary LN	1
Supraclavicular + axillary LN + inguinal LN	1
Supraclavicular + mediastinal + axillary + inguinal LN	1
Visceral organ metastasis only	
Bone	6
Lung	2
Bone + lung	2
Bone + liver	1
Visceral organ + distant LN metastasis	
Bone + inguinal LN	1
Bone + supraclavicular, inguinal LN	1
Lung + supraclavicular, inguinal LN	2

*Abbreviations*: *LN* lymph node.

The patients were divided into two groups according to the presence of visceral organ metastasis as follows: Group A, patients with distant LN metastasis only (n = 35) and Group B, patients with visceral organ metastasis (n = 15). Group A included more cases of squamous cell carcinoma than Group B (91.4% vs. 66.7%, p = 0.043). Patients in Group A received more EF-RT to cover regional LN than those in Group B (85.7% vs. 33.3%, p < 0.001). For Group A patients, median doses of 45 Gy (range, 36–54 Gy) were administered to the metastatic PALN, while a median dose of 59.4 Gy (range, 44–60 Gy) was administered to distant LNs. Other clinical characteristics and treatment profiles between Groups A and B did not differ significantly.

### Treatment response, patterns of failure and survival outcome

The median follow-up of surviving patients was 74 months (range, 24–296 months). The results of treatment response assessment in Group A are shown in Table 3. We found that 21 of the 35 patients achieved overall CR. There were a total of 35 failures among 50 patients (70.0%), and systemic progression was the dominant type of failure. Pelvic failures were found in six patients (12%), and systemic progression occurred in 32 patients (64%). Four patients had both locoregional failure and systemic progression.

**Table 3:** **Response after treatment in Group A (n = 35)**

	**CR (%)**	**PR (%)**	**SD (%)**	**PD (%)**
Primary cervical mass	29 (83)	5 (14)	1 (3)	0 (0)
Metastatic LN^a)^	22 (63)	12 (34)	0 (0)	1 (3)
Overall^b)^	21 (60)	12 (34)	1 (3)	1 (3)

*Abbreviations*: *CR* Complete remission, *PR* Partial remission, *SD* Stable disease, *PD* Progressive disease, *LN* lymph node.

^a)^Metastatic LN: Pelvic, paraaortic, supraclavicular, mediastinal, or axillary LN.

^b)^Overall: Treatment response in all lesions (Primary cervical mass + Metastatic LN).

**Figure 1:**
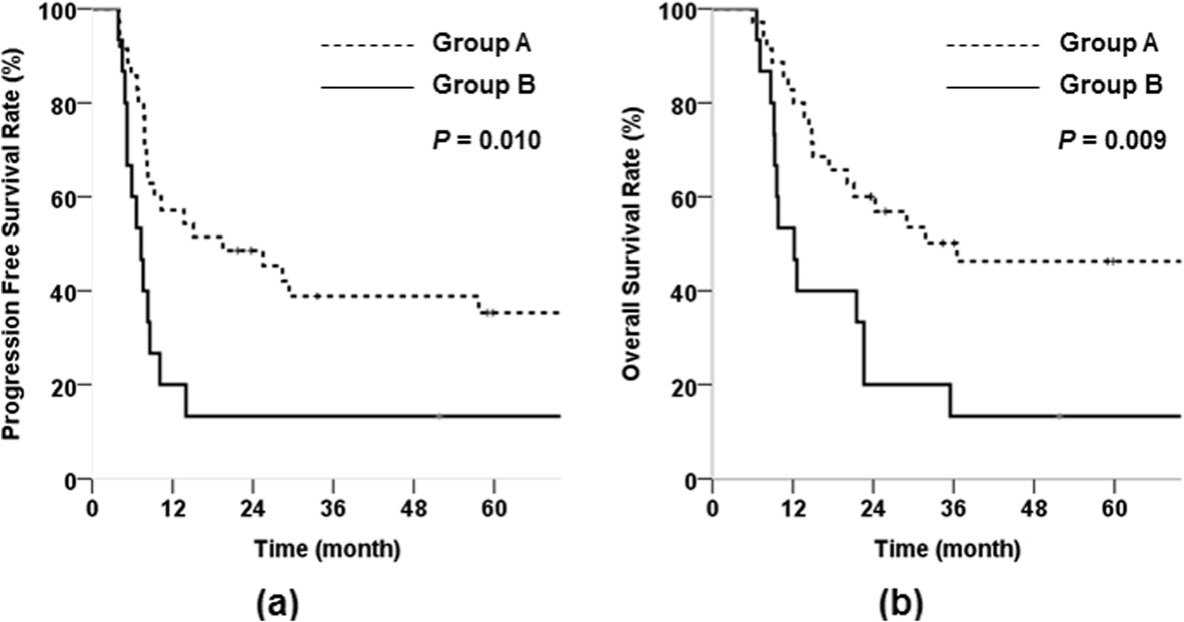
**Progression free survival rate (a), and overall survival rate (b) in group A and B (Group A = Distant LN metastasis only and Group B = Visceral organ metastasis).**

Eighteen of 50 patients (36%) survived until at least the end of the follow-up period. The median PFS and OS times were 9 and 23 months respectively. The 5-year PCR, PFS, and OS were 85.8%, 28.7%, and 36.2%, respectively. Univariate analysis was performed to identify significant prognostic factors affecting PCR, PFS, and OS. No significant prognostic factors affecting PCR were detected. Univariate analysis showed that tumor size > 4 cm and dissemination pattern were independent prognostic factors for PFS and OS (p < 0.05) (Table 4). The 5-year PFS and OS rates of Group A were significantly better than those of Group B (35.3% vs. 13.3%, p = 0.01; 46.3% vs. 13.3%, p = 0.009); the two groups had similar 5-year PCR (87.4% and 74.7%, respectively) (Figure 1). When patterns of failure were analyzed in terms of the two significant factors, some differences were observed: Compare to patients with small tumors, patients with large tumors were associated with more frequent pelvis recurrence than systemic progression. Group B more frequently exhibited systemic progression than Group A, despite a similar incidence of pelvis recurrence (Table 5).

**Table 4:** **Univariate and multivariate analysis of prognostic factors regarding progression free survival and overall survival**

**Prognostic factor**	**No. of patients**	**Univariate analysis**				**Multivariate analysis**			
		**5-y Survival rate (%)**				**PFS**		**OS**	
		**PFS**	**p-value**	**OS**	**p-value**	**HR (95% CI)**	**p-value**	**HR (95% CI)**	**p-value**
Age (y)			NS		NS	—	—	—	—
≤50	29	27.2		34.5					
>50	21	30.6		35.9					
Pathology			NS		NS	—	—	—	—
SCCa	42	27.5		33					
non-SCCa	8	33.3		50					
Pre-treatment Hb (g/dL)			NS		NS	—	—	—	—
≤10	16	18.8		18.8					
>10	34	32.9		44.6					
Tumor size (cm)			0.014		0.046		NS		NS
≤4	14	40.5		53.8		1		1	
>4	36	18.5		22.2		1.886 (0.777-4.577)		2.298 (0.839-6.292)	
Treatment type			NS		NS	—	—	—	—
RT + CTx	39	27.2		37.4					
RT alone	11	32.7		32.7					
Dissemination pattern			0.010		0.009		0.075		0.076
Group A	35	35.3		46.3		1		1	
Group B	15	13.3		13.3		1.954 (0.934-4.090)		1.962 (0.931-4.136)	

*Abbreviations*: *PFS* progression free survival, *OS* overall survival, *HR* hazard ratio, *NS* not significant, *SCCa* squamous cell carcinoma, *Hb* hemoglobin, *RT* Radiotherapy, *CTx* chemotherapy, *LN* lymph node, *Group A* Distant LN metastasis only, *Group B* Visceral organ metastasis.

**Table 5:** **The distribution of pattern of failures according to tumor size and patient group**

	**No. of patients**	**Pelvic recurrence**	**Systemic progression**
Tumor size (cm)			
≤4	14	1 (7.1%)	6 (42.9%)
>4	36	5 (13.9%)	26 (72.2%)
Dissemination pattern			
Group A	35	4 (11.4%)	20 (57.1%)
Group B	15	2 (13.3%)	12 (80.0%)

*Abbreviations*: *LN* lymph node, *Group A* Distant LN metastasis only, *Group B* Visceral organ metastasis.

**Figure 2:**
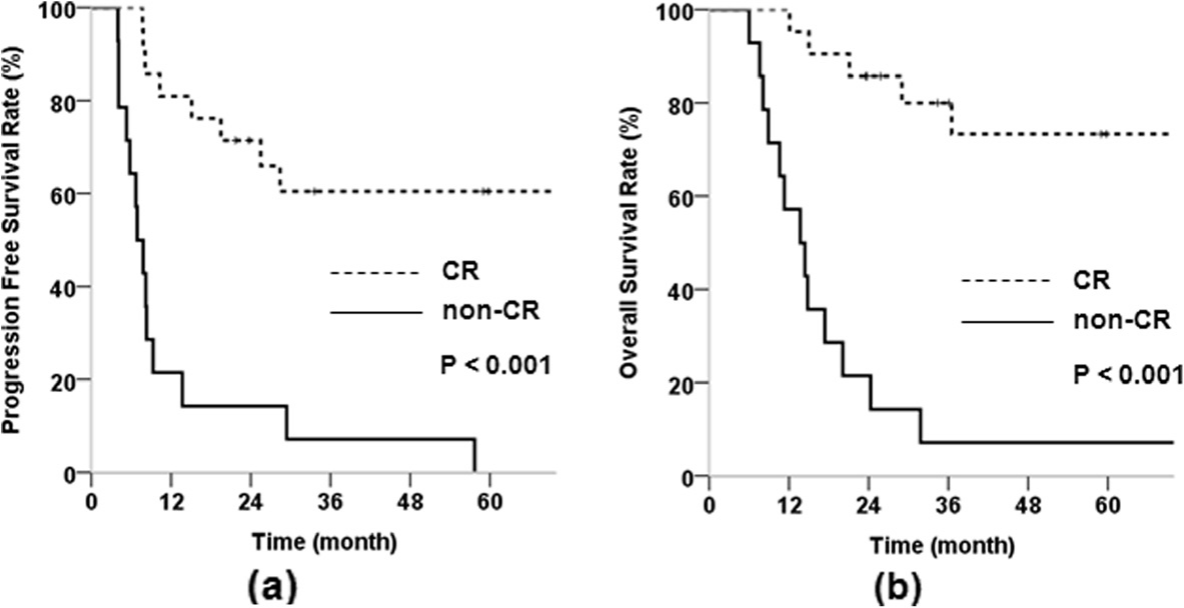
**Comparison of survival curves according to treatment response (complete remission (CR) vs. non-CR) in Group A. (a)** Progression free survival rate, **(b)** overall survival rate.

The results of multivariate analysis of PFS and OS and are shown in Table 3. None of the prognostic factors showed an association with PFS and OS on multivariate analysis. However, patients with large tumors showed an approximately two-fold increased risk of tumor progression compared to patients with small tumors. As well, Group B patients exhibited an approximately two-fold increased risk of progression and death compared to those in Group A, with marginal significance (HR, 1.954 and 1.962, respectively). In Group A, we evaluated the effect of CR on PFS and OS rates. (Figure 2); the 21 patients who achieved overall CR had significantly better 5-year PFS and OS rates than the 14 non-CR patients (60.4% vs. 0%, p < 0.001; 73.3% vs. 7.1%, p < 0.001).

## Discussion

Owing to the recent advances in imaging technology, disseminated disease is encountered more frequently during the management of cervical cancer patients. However, FIGO staging does not differentiate between different types of metastatic spread, and no consensus has been reached in terms of standardized treatment for disseminated disease. In fact, the National Comprehensive Cancer Network Guidelines only offer treatment recommendations for distant metastasis [18]; two treatment plans have been suggested according to whether the disease is amenable to local treatment. Nevertheless, criteria for amenability to local treatment have also not been defined. Therefore, determining which patients should receive local treatment warrants investigation. As most disseminated cases present with locally advanced disease, the decision to use RT rather than surgery would have a significant influence on prognosis. In addition, as FIGO classification does not provide nodal staging for cervical cancer, no separate guidelines have been established to treat nodal metastasis. Usually, radiation oncologists design radiation fields to cover regional nodal metastases for the whole pelvis or EF. However, patients with LN metastasis beyond the extended field, such as in the mediastinal, axillary, or supraclavicular area, are considered to have disseminated disease in accordance with stage IVB.

The prognoses of disseminated disease are heterogeneous, and dependent on the distribution of the dissemination and tumor bulk [12,13]. Some recent studies have shown that aggressive treatment using CCRT is safe and effective for patients with limited LN metastasis, such as SCLN metastasis [7-9,19,20]. Kim et al. reported that the 3-year OS rate in patients with PALN and SCLN metastases was 49%, when treated with curative CCRT [7]. They suggested that CCRT may be more effective than systemic CTx for improving survival in stage IVB cervical cancer patients with distant lymphatic metastasis [9]. However, patients with visceral organ metastasis were associated with a worse prognosis due to systemic progression.

A personalized RT approach, based on the status of visceral organ metastasis, may benefit patients with disseminated cervical cancer. In patients with lymphatic metastasis without visceral organ metastasis, definitive RT with CTx might be recommended to achieve maximal local control and survival benefit. Several investigators reported that overall CR was achieved in 50 ~ 60% patients treated with CCRT, including those with distant lymphatic metastasis [7,9]. Our findings of overall CR in 60% of patients with distant LN were comparable to the results of these two recent retrospective studies. Nevertheless, only systemic CTx was applied to patients with distant lymphatic metastasis in one of these studies, inducing CR in 0% of the patients [9]. In patients with uterine cervical carcinoma with PALN metastasis, treatment response was the only statistically independent prognostic factors for OS [21]. The present study also discerned that overall CR is an important prognostic factor for 5-year OS rate. Therefore, implementing RT concurrently with CTx, rather than systemic CTx alone, may be beneficial to inducing overall CR. To achieve maximum local control for these patients, EBRT on metastatic LNs in addition to pelvic RT with ICR might be recommended.

Even though high pelvic controls rates were achieved in patients with visceral organ metastasis treated with definitive RT, the survival outcome was dependent on progression of visceral organ metastasis. Therefore, the importance of systemic CTx in delaying systemic progression should be emphasized. Recently, new cytotoxic CTx and molecular targeted agents have been investigated for the treatment of recurrent or metastatic disease: Paclitaxel-based CTx has been shown to have a radiosensitizing effect with objective response rates of 33–67.9% for carboplatin/paclitaxel studies and 29.1-67% for cisplatin/paclitaxel studies [22]; Cisplatin/paclitaxel CTx was found to be the best combination for the treatment of advanced or recurrent cervical cancer [3]. The overall response rate of combined docetaxel, carboplatin, and 5-fluorouracil in patients with metastatic cervical carcinoma was 56% [23]. The response rate was 48% among patients with recurrent, persistent, or metastatic cervical cancer who received bevacizumab and cisplatin/paclitaxel CTx [24]. CCRT and adjuvant gemcitabine/cisplatin CTx in patients with stage IIB to IVA disease improved survival outcomes when compared with CCRT [25]. CCRT followed by adjuvant gemcitabine/cisplatin CTx may also benefit for disseminated uterine cervical cancer patients. In clinical practice, we have recently observed some patients with good responses after upfront systemic CTx prior to RT, even though they are not included in this analysis. Despite a lack of supporting evidence, upfront systemic CTx might be a useful option for patients with visceral metastasis. If the response after chemotherapy is favorable, RT might be extended with the consolidation aim of controlling residual disease following CTx. Otherwise, the role of RT might be limited to palliative therapy to relieve vaginal bleeding and pelvic pain, or to prevent vesicovaginal or rectovaginal fistula caused by local progression. Palliative RT is effective for providing relief from vaginal bleeding, pain, and other symptoms [4,5,26]. The overall response rate was more than 90% for vaginal bleeding control [5,26]. The present study also revealed that the 5-year PCR is 85.8%. Pelvic RT might be beneficial to palliate symptom and delay pelvic progression in patients with visceral organ metastasis.

The current study has several limitations. Since the present study is a retrospective review covering a long period, heterogeneity of patient characteristics might have confused treatment, follow-up, and results. Heterogeneous treatments might be a confounding factor. Also, unrecognized biases could not be considered in this study. Because of the low incidence of this disease, a small number of patients enrolled. Compared to the previous institutional studies, however, this study had the greatest number of patients [11,12].

## Conclusion

Notwithstanding the inherent drawbacks of a retrospective analysis, our data suggests the application of personalized radiotherapeutic strategies for patients presenting with disseminated cervical cancer. Definitive RT, including pelvis and affected distant nodal metastasis, may be more beneficial to patients with disseminated cervical cancer limited to distant LN metastasis only. Meanwhile, in patients with visceral organ metastasis, systemic CTx might be considered to delay systemic progression, while administering RT to relieve local symptoms and to delay pelvic progression.

## Abbreviations

RT: Radiotherapy
PCR: Pelvic control rates
PFS: Progression free survival
OS: Overall survival
FIGO: International Federation of Gynecology and Obstetrics
LN: Lymph node
EF: Extended field
CTx: Chemotherapy
CCRT: Concurrent chemoradiotherapy
SCLN: Supraclavicular lymph node
EBRT: External beam radiation therapy
ICR: Intracavitary irradiation
CR: Complete remission
HR: Hazard ratios
CI: Confidence interval
PALN: Para-aortic lymph node
